# A simple risk stratification model that predicts 1-year postoperative mortality rate in patients with solid-organ cancer

**DOI:** 10.1002/cam4.518

**Published:** 2015-08-26

**Authors:** Wen-Chi Chou, Frank Wang, Yu-Fan Cheng, Miao-Fen Chen, Chang-Hsien Lu, Cheng-Hsu Wang, Yung-Chang Lin, Ta-Sen Yeh

**Affiliations:** 1 Department of Medical Oncology, Chang Gung Memorial Hospital at LinKouTaoyuan, Taiwan; 2 Graduate Institute of Clinical Medical Sciences, College of Medicine, Chang Gung UniversityTaoyuan, Taiwan; 3 Departments of Surgery, Chang Gung Memorial Hospital at LinKouTaoyuan, Taiwan; 4 Department of Surgery, School of Medicine, University of SydneySydney, Australia; 5 Department of Radiology, Chang Gung Memorial Hospital at KaoshiungKaoshiung, Taiwan; 6 Department of Radiation Oncology, Chang Gung Memorial Hospital at ChiayiChiayi, Taiwan; 7 Department of Medical Oncology, Chang Gung Memorial Hospital at ChiayiChiayi, Taiwan; 8 Department of Medical Oncology, Chang Gung Memorial Hospital at KeelungKeelung, Taiwan

**Keywords:** Postoperative mortality, prognostic score, risk model, solid cancer, validation

## Abstract

This study aimed to construct a scoring system developed exclusively from the preoperative data that predicts 1-year postoperative mortality in patients with solid cancers. A total of 20,632 patients who had a curative resection for solid-organ cancers between 2007 and 2012 at Chang Gung Memorial Hospital Linkou Medical Center were included in the derivation cohort. Multivariate logistic regression analysis was performed to develop a risk model that predicts 1-year postoperative mortality. Patients were then stratified into four risk groups (low-, intermediate-, high-, and very high-risk) according to the total score (0–43) form mortality risk analysis. An independent cohort of 16,656 patients who underwent curative cancer surgeries at three other hospitals during the same study period (validation cohort) was enrolled to verify the risk model. Age, gender, cancer site, history of previous cancer, tumor stage, Charlson comorbidity index, American Society of Anesthesiologist score, admission type, and Eastern Cooperative Oncology Group performance status were independently predictive of 1-year postoperative mortality. The 1-year postoperative mortality rates were 0.5%, 3.8%, 14.6%, and 33.8%, respectively, among the four risk groups in the derivation cohort (c-statistic, 0.80), compared with 0.9%, 4.2%, 14.6%, and 32.6%, respectively, in the validation cohort (c-statistic, 0.78). The risk stratification model also demonstrated good discrimination of long-term survival outcome of the four-tier risk groups (*P* < 0.01 for both cohorts). The risk stratification model not only predicts 1-year postoperative mortality but also differentiates long-term survival outcome between the risk groups.

## Introduction

Comprehensive preoperative assessment and accurate prediction of surgery-related risks are vital in ensuring better patient selection and improved surgical outcomes in the management of solid-organ cancers. Postoperative mortality is one of the most used study endpoints in the literature that indicates the quality of cancer surgery and postoperative care. Presently, 30-day and in-hospital mortality rates for various cancer types are well documented in institutional 1–5 and national statistics 6,7. Using the Dutch Cancer Registry, Damhuis et al. showed that 43% of in-hospital deaths for esophageal cancer occurred 30 days or later after the operation 8. Extending the mortality period beyond 30 days has the advantage that patients who die of surgery-related complications outside the hospital are also included in the database. Indeed, recent literature suggests that extending postoperative mortality beyond 30 days and longer would better indicate the true incidence of surgery-related mortality and quality of surgical care 9–12. The ability to predict postoperative mortality risk before surgery provides valuable prognostic information to the clinicians, the patients, and their families 13,14. The aim of this study was to develop and validate a simple risk scoring model using exclusively preoperative data that predicts 1-year postoperative mortality for patients with different solid-organ cancers.

## Patients and Methods

### Patient selection

A retrospective analysis was performed on patients who underwent operations for primary solid-organ cancers between January 2007 and December 2012 at Chang Gung Memorial Hospital (CGMH) Linkou Medical Center (derivation cohort). Patients with either pathologically or radiologically proven malignancies undergoing a curative resection for the primary cancer were included in the study. Patients who received a palliative procedure (resections or bypass surgery) were excluded. In addition, patients with skin cancers and superficial urinary bladder cancers were also precluded. The validation cohort consisted of a group of consecutively operated cancer patients with the same selection and exclusion criteria who were enrolled over the same study period from the three CGMH affiliated hospitals (Keelung branch, regional hospital; Chiayi branch, regional hospital; and Kaohsiung branch, medical center). The study was approved by the Institutional Review Board in all CGMH branches.

### Data collection

The prospectively collected administrative and clinical data included patient demographics, American Society of Anesthesiologist score (ASA score), and Charlson comorbidity index (CCI). Patient demographics, including age, gender, Eastern Cooperative Oncology Group performance status (ECOG scale), history of previous cancer, preexisting comorbidities, cancer by anatomic location, and clinical tumor staging, were recorded prospectively by the primary care clinicians using an electronic patient record form. The electronic patient record form was introduced in2006 by the institutional cancer center with the intention to improve the quality of cancer patient care after the implementation of Cancer Prevention and Treatment Act in Taiwan. The clinical data were completed and maintained by the individual multidisciplinary cancer care teams and the cancer center. Tumor stage was recorded as localized, regional, advanced, and unclassified using SEER summary stage classification 14. American Society Anesthesiologist scores were evaluated by the anesthesiologists at preanesthetic evaluation, while CCIs were calculated from the electronic patient record forms using International Classification of Diseases, Ninth Revision (ICD-9). Patient who had a diagnosis of more than one cancer or had received more than one operation for the primary tumor during the study period was analyzed from the date of operation for the first tumor or the first surgery.

### Follow-up

All patients were followed up until death or 30 June 2014. Survival time was determined from the time of surgery to death or 30 June 2014. The date of death was obtained from the National Registry of Death database in Taiwan. The incidences and the mortality rates of cancers were obtained from the Taiwan Cancer Registry (TCR).

### Statistical analysis

Patient demographic data were summarized as numbers and percentages for categorical variables, and medians and interquartile ranges (IQRs) for continuous variables. The following nine variables were assessed in the univariate logistic regression model (with a binary outcome of death or survival at 1 year after operation): gender, age (decades), cancer type, history of previous cancer, tumor stage, ASA score, ECOG scale, admission type (elective or nonelective), and CCI. The optimal category of variables was selected in order to generate preliminary data with better discrimination of mortality rates. Tests for interaction among the nine variables were performed using interaction terms in a multivariate logistic regression model. This model was then used to determine independent predictors of 1-year postoperative mortality and their odds ratios (ORs). The *β*–coefficients from the final model were used to generate point scores for the 1-year postoperative mortality risk. Patients were then stratified into four risk groups according to the total score obtained from the mortality risk analysis using a Cox regression model. Receiver operating characteristic (ROC) curves and the area under the curve (c-statistic) were used to determine the accuracy of the 1-year postoperative mortality risk model. A c-statistic >0.70 was considered as a reasonable model. The validity of the resulting mortality risk model was then assessed by the validation cohort. Overall survival among the four risk groups was estimated by the Kaplan–Meier method, and the differences in survival distributions were analyzed by the Log-rank test. SPSS 17.0 software (SPSS Inc., Chicago, IL) was used for all statistical analyses. A *P* < 0.05 was considered to be statistically significant.

## Results

### Patient characteristics

A total of 20,632 and 16,656 cancer patients who underwent a curative resection for primary solid-organ cancers were included in the derivation and validation cohorts, respectively. Demographic characteristics were similar between the derivation and validation cohorts (Table1). All primary cancers were initially grouped into 12 types according to the anatomic locations. These were colorectal, head and neck, breast, gynecologic, genitourinary, hepato–biliary–pancreatic (HPB), stomach and small bowel, thyroid, thorax, central nervous system (CNS), esophagus, and others. The median follow-ups were 39.5 months (IQR, 23.3–60.9 months) and 38.1 months (IQR, 22.0–59.9 months) in the derivation and validation cohorts, respectively. The 1-year postoperative mortality rates were 9.4%, 10.4%, and 9.8% in the derivation, validation, and overall cohorts, respectively.

**Table 1:** Patient’s preoperative demographic data

Variable	Categories	Derivation cohort		Validation cohort		Overall	
		*N*	%	*N*	%	*N*	%
Total		20,632	100	16,656	100	37,288	100
Gender	Male	10,613	51.4	9047	54.3	19,660	52.7
Age	Median, IQR	57	48–68	59	50–69	58	49–68
	Colorectal	4766	23.1	4405	26.4	9171	24.6
	Head and neck	3415	16.6	2751	16.5	6166	16.5
	Breast	2364	11.5	1579	9.5	3943	10.6
	Gynecologic	2198	10.7	1366	8.2	3564	9.6
	Genitourinary tract	1922	9.3	1604	9.6	3526	9.5
	Hepato–pancreatic	1760	8.5	1859	11.2	3619	9.7
Primary cancer site	Stomach, small bowel	1311	6.4	899	5.4	2210	5.9
	Thyroid	1069	5.2	770	4.6	1839	4.9
	Thorax	774	3.8	768	4.6	1542	4.1
	Central nervous system	466	2.3	223	1.3	689	1.8
	Esophagus	246	1.2	233	1.4	479	1.3
	Others	341	1.7	199	1.2	540	1.4
Previous cancer history	Yes	2190	10.6	2109	12.7	4299	11.5
Tumor stage	Localized	4193	20.3	2779	16.7	6972	18.7
	Regional	6438	31.2	5064	30.4	11,502	30.8
	Advanced	6023	29.2	5215	31.3	11,238	30.1
	Unclassified	3978	19.3	3598	21.6	7576	20.3
	0	8646	41.9	6494	39.0	15,140	40.6
	1	8548	41.4	6569	39.4	15,051	40.4
ECOG scale	2	2441	11.8	2576	15.5	4981	13.4
	3	908	4.4	913	5.5	1882	5.0
	4	89	0.4	104	0.6	234	0.6
Admission type	Elective	18,239	88.4	13,915	83.5	32,154	86.2
	Nonelective	2393	11.6	2741	16.5	5134	13.8
CCI	0	14,900	72.2	10,849	65.1	25,749	69.1
	1	4191	20.3	3795	22.8	7986	21.4
	2	1056	5.1	1346	8.1	2402	6.4
	3	286	1.4	401	2.4	687	1.8
	4	126	0.6	173	1.0	299	0.8
	≥5	73	0.4	165	1.0	238	0.6
ASA score	1	1418	6.9	1510	9.1	2929	7.9
	2	12,476	60.5	10,144	60.9	22,620	60.7
	3	6694	32.4	4907	29.5	11,601	31.1
	4 and 5	44	0.2	95	0.6	139	0.4
One-year postoperative mortality		1930	9.4	1728	10.4	3658	9.8

ASA, American Society of Anesthesiologist; CCI, Charlson comorbidity index; IQR, interquartile range; ECOG, Eastern Cooperative Group Oncology.

### Longitudinal postoperative mortality rates of the derivation cohort

The 1-, 3-, 6-months, and 1-year postoperative mortality rates of the derivation cohort, as stratified by the study variables, are shown in Table2. Of note, the 12 anatomy-based cancer types were reclassified into five cancer groups according to their potential postoperative mortality risk. The 1-year postoperative mortality rates were significantly higher in male gender, oldest old (>80 year-old), digestive tract cancers, CNS cancers, advanced tumor stage, poor ECOG scale, nonelective admission, and poor CCI, in comparison with the mean 1-year postoperative mortality rate of the whole derivation cohort.

**Table 2:** Longitudinal postoperative mortality of cancer patients after radical resections

		Postoperative mortality rate (%)			
Variable	Category	1-month	3-months	6-months	1-year
Total		0.7	2.2	4.4	9.4
Gender	Female	0.6	1.6	3.2	6.7
	Male	0.9	2.7	5.5	11.8
Age, years	0–69	0.5	1.4	3.2	7.4
	70–79	1.3	4.0	7.2	14.2
	≥80	2.9	7.7	11.8	23.2
Primary tumor site	Breast, thyroid	0.2	0.6	1.0	1.7
	CRC, GYN, and GU	0.7	1.8	3.4	7.1
	HN, Esophagus, thorax, and others	0.5	2.0	4.8	12.7
	HPB, stomach, and small bowel	1.7	5.0	9.3	17.2
	CNS	1.9	5.2	10.3	22.5
Previous cancer history	No	0.7	2.1	4.1	8.9
	Yes	0.9	3.1	6.3	13.0
Tumor stage	Localized	0.5	1.2	2.0	4.0
	Regional	0.8	2.5	5.7	12.8
	Advanced	2.5	7.2	14.2	28.8
	Unclassified	1.1	3.4	5.0	9.3
ECOG scale	0–1	0.6	1.6	3.4	7.4
	2–3	1.5	4.8	8.9	18.5
	4	9.0	16.9	25.8	38.2
Admission type	Elective	0.5	1.5	3.3	7.4
	Nonelective	2.6	7.1	12.7	23.9
CCI	0	0.3	1.2	3.1	7.4
	1–2	1.1	3.2	6.2	12.7
	3–4	8.0	14.8	18.7	26.9
	≥5	24.7	49.3	57.5	60.3
ASA score	1–2	0.3	1.1	2.6	6.1
	3–5	1.6	4.3	8.1	16.0

ASA, American Society of Anesthesiologist; CCI, Charlson comorbidity index; ECOG, Eastern Cooperative Oncology Group; HPB, hepato–biliary–pancreatic; CNS, central nervous system.

### Independent predictive factors of 1-year postoperative mortality in the derivation cohort

The results of the univariate and multivariate analyses are shown in Table3. Age, gender, cancer group, history of previous cancer, tumor stage, CCI, ASA score, admission type, and ECOG scale were all independent variables influencing 1-year postoperative mortality.

**Table 3:** Univariate and multivariate analysis for 1-year postoperative mortality in derivation cohort

Variable	Categories	*N*	No. of 1-year postoperative mortality (%)	Univariate			Multivariate		
				OR	95% CI	*P*	OR	95% CI	*P*
Gender	Female	10,019	676 (6.7)	1			1		
	Male	10,613	1254 (11.8)	1.85	1.68–2.04	<0.001	1.17	1.04–1.31	0.008
Age	0–69	16,138	1192 (7.4)	1			1		
	70–79	3383	480 (14.2)	2.07	1.85–2.32	<0.001	1.57	1.38–1.80	<0.001
	≥80	1111	258 (23.2)	3.79	3.26–4.41	<0.001	2.67	2.23–3.21	<0.001
Primary cancer site	Breast or thyroid	3433	60 (1.7)	1			1		
	CRC, GYN, and GU	8886	632 (7.1)	4.3	3.29–5.63	<0.001	2.06	1.55–2.74	<0.001
	HN, Esophagus, Lung, and others	4776	605 (12.7)	8.15	6.23–10.7	<0.001	6.53	4.88–8.72	<0.001
	HPB, stomach, and small bowel	3071	528 (17.2)	11.7	8.89–15.3	<0.001	5.48	4.08–7.35	<0.001
	CNS	466	105 (22.5)	16.4	11.7–22.9	<0.001	14.3	9.94–20.6	<0.001
Previous cancer history	No	18,442	1645 (8.9)	1			1		
	Yes	2190	285 (13.0)	1.53	1.34–1.75	<0.001	1.54	1.33–1.790	<0.001
Tumor stage	Localized	10,329	418 (4.0)	1					
	Regional	8194	1049 (12.8)	3.48	3.09–3.92	<0.001	3.74	3.30–4.24	<0.001
	Advanced	1366	394 (28.8)	9.61	8.25–11.2	<0.001	13.8	11.6–16.4	<0.001
	Unclassified	743	69 (9.3)	2.43	1.86–3.17	<0.001	2.99	2.23–4.00	<0.001
ECOG scale	0–1	17,194	1278 (7.4)	1			1		
	2–3	3349	618 (18.5)	2.82	2.54–3.13	<0.001	1.47	1.30–1.67	<0.001
	4	89	34 (38.2)	7.69	5.00–11.8	<0.001	2.63	1.60–4.31	<0.001
Admission type	Elective	18,239	1358 (7.5)	1			1		
	Nonelective	2393	572 (23.9)	3.91	3.50–4.35	<0.001	2.04	1.79–2.32	<0.001
CCI	0	14,900	1107 (7.4)	1			1		
	1–2	5247	668 (12.7)	1.82	1.64–2.01	<0.001	1.23	1.10–1.38	<0.001
	3–4	412	111 (26.9)	4.60	3.67–5.76	<0.001	2.66	2.04–3.47	<0.001
	≥5	73	44 (60.3)	18.9	11.8–30.3	<0.001	9.43	5.45–16.3	<0.001
ASA score	1–2	13,894	850 (6.1)	1			1		
	3–5	6738	1080 (16.0)	2.93	2.66–3.22	<0.001	1.44	1.29–1.62	<0.001

ASA, American Society of Anesthesiologist; CCI, Charlson comorbidity index; ECOG, Eastern Cooperative Oncology Group; HPB, hepato–biliary–pancreatic; CNS, central nervous system.

### Risk model, risk group classification and model accuracy in the derivation cohort

**Figure 1:**
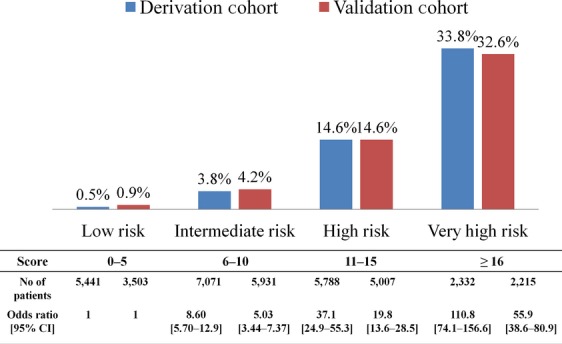
One-year postoperative mortality rates in cancer patients of derivation and validation cohorts stratified by four-tier risk potential.

**Figure 2:**
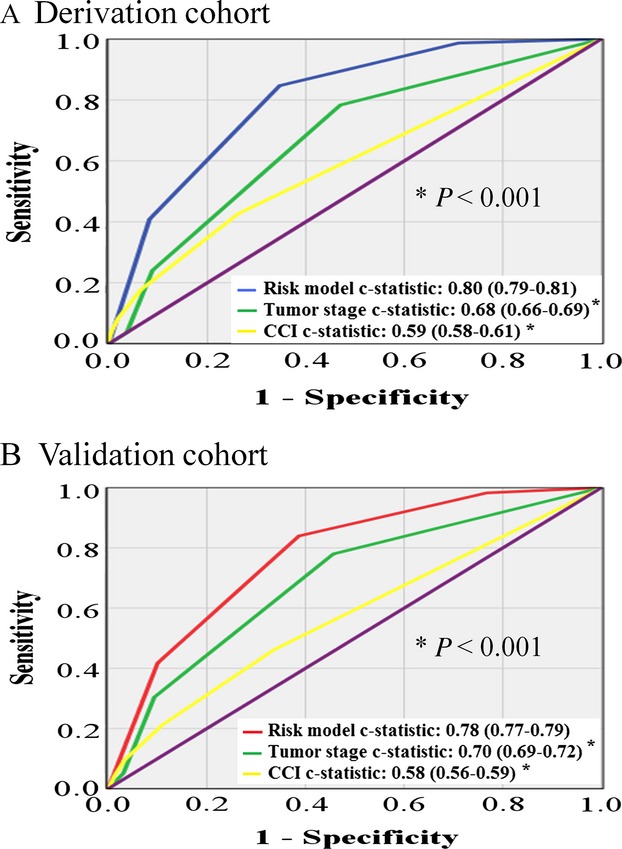
Area under curve for 1-year postoperative mortality in cancer patients of derivation (A) and validation (B) cohort constructed by risk model, tumor stage, and Charlson comorbidity index, respectively. Notably, all *P* < 0.01 for comparisons among three curves.

The 1-year postoperative mortality risk model and the scoring system generated from the *β*–coefficients of the multivariate analysis are summarized in Table4. Patients were stratified into low-, intermediate-, high-, and very high-risk groups on the basis of the total-risk model score; and the corresponding 1-year postoperative mortality rates and the ORs of the four risk groups in the derivation cohort were shown (Fig.1). The c-statistic of the four-tier risk model was up to 0.80 (95% confidence interval [CI], 0.79–0.81); whereas the c-statistics were decreased to 0.68 and 0.64, when constructed by tumor stage and CCI, respectively (Fig.2A).

**Table 4:** Score calculation to predict 1-year postoperative mortality in derivation cohort

Variable	Categories	*β*-coefficient (SE)	*P*-value	Point score
Gender	Female (reference)	0		0
	Male	0.06 (0.02)	0.008	1
Age	0–69 (reference)	0		0
	70–79	0.17 (0.03)	<0.001	2
	≥80	0.37 (0.03)	<0.001	4
Primary cancer site	Breast, thyroid (reference)	0		0
	CRC, GYN, and GU	0.28 (0.05)	<0.001	3
	HN, Esophagus, Lung, and others	0.71 (0.06)	<0.001	7
	HPB, stomach, and small bowel	0.62 (0.06)	<0.001	6
	CNS	1.0 (0.07)	<0.001	10
Previous cancer history	No (reference)	0		0
	Yes	0.17 (0.03)	<0.001	2
Tumor stage	Localized (reference)	0		0
	Regional	0.50 (0.02)	<0.001	5
	Advanced	0.99 (0.03)	<0.001	10
	Unclassified	0.41 (0.06)	<0.001	4
ASA score	1–2 (reference)	0		0
	3–5	0.13 (0.02)	<0.001	1
ECOG scale	0–1 (reference)	0		0
	2–3	0.13 (0.02)	<0.001	1
	4	0.36 (0.09)	<0.001	4
Admission type	Elective (reference)	0		0
	Nonelective	0.28 (0.02)	<0.001	3
CCI	0 (reference)	0		0
	1–2	0.08 (0.02)	<0.001	1
	3–4	0.37 (0.05)	<0.001	4
	≥5	0.83 (0.10)	<0.001	8
Total score			0–43	

ECOG: Eastern Cooperative Oncology Group; SE: standard error.

### Validation of risk model accuracy for 1-year postoperative mortality

In the validation cohort, the 1-year postoperative mortality rates were 0.9%, 4.2%, 14.6%, and 32.6% for the low-, intermediate-, high-, and very high-risk groups, respectively, which were comparable to those of the derivation cohort (Fig.1). Again, the c-statistics of these four-tier risk model was 0.78 (95% [CI], 0.77–0.79); whereas the c-statistics were decreased to 0.70 and 0.60, when constructed by tumor stage and CCI, respectively (Fig.2B).

### Survival outcomes of the derivation and validation cohorts

**Figure 3:**
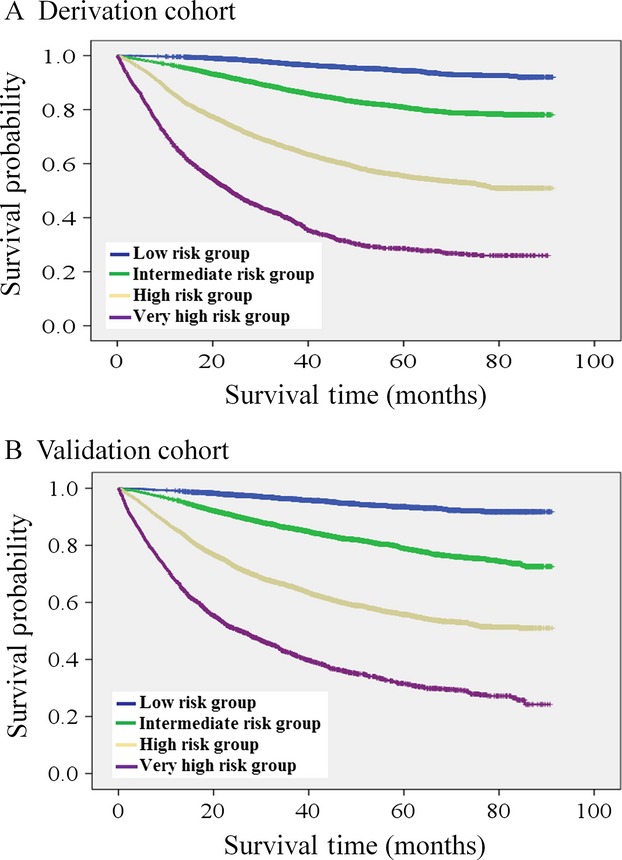
Kaplan–Meir survival curves of cancer patients among derivation cohort (A) and validation cohort (B) stratified by four-tier risk potential.

At the date of study censor, 9.4% of patients in the derivation cohort and 10.4% of patients in the validation cohort died 1 year after surgery. Statistically significant survival differences out to 80 months postoperatively were observed between the four risk model groups in both derivation (*P* < 0.001) and validation cohorts (*P* < 0.001) (Fig.3, Table5).

**Table 5:** Risk group by score calculation in derivation cohort

Risk group	Sum score	No. of patient	No. of 1-year postoperative mortality (%)	Odds ratio	95% CI	*P*-value
Low risk	0–5	5441	25 (0.5)	1		
Intermediate risk	6–10	7071	270 (3.8)	8.60	5.70–12.9	<0.001
High risk	11–15	5788	846 (14.6)	37.1	24.9–55.3	<0.001
Very high risk	≥16	2332	789 (33.8)	110.8	74.1–156.6	<0.001

## Discussion

In the present study, we audited the longitudinal mortality rates on a large cohort of patients with solid-organ cancers who had undergone a curative resection in a tertiary referral medical center within a 6-year period. Of the solid-organ cancers identified lung, liver, colorectal, breast, head and neck, prostate, gastric, pancreatic, esophageal, and gynecological cancers, in the order of decreasing prevalence, represent the ten leading causes of cancer-related death in Taiwan 15. Through the integration of the prospectively collected electronic medical records 16, administrative data such as ICD-9, and deceased data from TCR 15, we were able to analyze patient demographics, cancer types, history of previous cancer, tumor stage, admission type, and associated medical comorbidities in a nonbiased and quantitative manner with minimal missing data. By correlating these preoperative data with the 1-year postoperative mortality rates, a simple, point scoring, and risk stratification tool was developed using multivariate logistic regression method. The derivative risk model was then validated by an independent patient cohort and shown to be functioning well by the ROC (receiver operation characteristic) curves.

In an era of advancing scientific knowledge and technology, there is a growing demand from the cancer patients and their treating clinicians for a simple risk stratification model that could accurately predict postoperative mortality and allow for better patient selection to avoid under and over treatment, and substantially improve survival outcomes and life quality 17–20. The mortality risk model was developed entirely from the preoperative data without taking into account of specific laboratory results, intraoperative variables, and immediate postoperative complications. Factors that are commonly cited in a risk-adjusted predictive model for cancer patient outcomes include age, gender, cancer site, tumor staging, laboratory data, intraoperative findings, and postoperative complications. However, functional status of a patient, an important factor in determining the treatment outcome, is not routinely collected in the administrative datasets. ECOG scale, ASA score, and CCI, either alone or in combination, are the three commonly used measurements that reflect the functional status of a patient 21–26. Although other investigators have examined the predictive value of these functional measurements for postoperative morbidity and mortality in the surgical treatment of cancer patients at various time points such as 30, 60, or 90 days 19,25–29, the results have been mixed and none has indicated an optimal combination. In this study, ECOG scale, ASA score, and CCI have all been shown to independently influence 1-year postoperative mortality in our cohort of cancer patients.

In this study, the 12anatomy -based cancer types were reallocated into five cancer groups according to their observed 1-year postoperative mortality. As illustrated in Table2, the highest 1-year postoperative mortality rate was observed in CNS surgery, followed by HPB and upper gastrointestinal tract, head and neck and lung, colorectal and genitourinary, and breast and thyroid surgeries, in descending order. This organ-specific postoperative risk classification is imperative for two reasons. Firstly, it serves to indicate the radicality of the surgical procedures that involves removal of vital organs and reduction in their functional reserve such as cognition, digestion, and respiration. Secondly, it partly reflects the life expectancy that is inherently linked to the biology of individual cancer. As a result, the probability of 1-year postoperative mortality in colorectal, esophageal, HPB, and brain cancer patients were 2.0-, 6.5-, 5.4-, and 14.3-fold, respectively, in comparison with that of breast and thyroid cancers in this study.

The available evidence would suggest that elderly cancer patients tend to have a higher rate of postoperative morbidity, either procedure-related or unrelated, than the younger counterparts 17,19,20. In a national population-based outcome study, Finlayson et al. reported that operative mortality among the octogenarians was higher than that of the younger patients for the three major cancer surgeries investigated, namely, esophagectomy, pancreatectomy, and lung resection 6. In addition, long-term survival among octogenarians with two or more comorbidities was worse than those with fewer comorbid diagnoses. Similarly, our recent publication also demonstrated a higher surgical morbidity rate in the older group of patients with gastric cancer (>80 years) than in the younger group (<80 years, 18.3% vs. 12.6%, *P* = 0.035) 16. Although the 30-day mortality rates did not differ significantly between the two groups, the in-hospital mortality rate of the older group was higher than that of the younger group (6.7% vs. 3.1%, *P* = 0.016). These findings suggested that the elderly patients were more likely to succumb to postoperative complications because of preexisting or unrecognized comorbidities. Robinson et al. have shown that the constellation of frailty, disability, and comorbidity were associated with 6-month mortality rate up to 15% in elderly patients following general, thoracic, vascular, and urologic procedures 18. Based on the risk model developed in the present study, a male octogenarian with regional-staged gastric cancer would be stratified to the very high-risk group, even without any medical comorbidity, and the predicted 1-year postoperative mortality rate would be as high as 33.8%. This prediction is astonishingly higher than our previously reported 30-day and in-hospital mortality rates of 3.0% and 6.7%, respectively 16. The discrepancy in the mortality rates highlights the importance of appropriate patient selection, preoperative risk stratification, and refinement of postoperative care.

Undoubtedly, some might argue that 1-year postoperative mortality links with tumor factors and patients’ factors; thus it should distinguish 1-year relapse-free mortality from cancer-specific mortality. According to our data, tumor staging accounted for the most influencing component among our nine independent variables, up to 10-point score for advanced stage in our scoring system. Advanced tumor stage always indicate more aggressive surgical extent and more devastating loss of functional reservoir, as well higher probability of early cancer recurrence. Taken together, our 1-year postoperative mortality would be relevant to either sequel of surgical complications or early cancer recurrence or both. In many circumstances, the cause of death was not easily elucidated. Nevertheless, from practical point of view, overall postoperative mortality at 1 year represents our major concern rather than relapse-free mortality. Regarding to this perspective, our integrated risk model provided a much better power (c-statistic, 0.80) to predict 1-year postoperative mortality, compared with that by either tumor factor (tumor stage, c-statistic, 0.68) or patients’ factor (CCI, c-statistic, 0.59) alone.

Lastly but not least importantly, our risk stratification model additionally conferred good discrimination and accuracy of long-term survival of four-tier risk groups irrespective of their original cancer types and staging, even though our initial proof-of-concept was not designed for this purpose. Similar to that of 1-year postoperative mortality, long-term survivals of cancer patients after definite treatment are in large extent determined by the cancer types, tumor stage, and patients’ factors such as age, nutrition, comorbidity, and frailty, which have been incorporated into our risk stratification model. Notwithstanding, the high- or very high-risk group cancer patients usually underwent less intensively radical surgery than those of standard patients, and less frequently receive adjuvant chemotherapy, as well more often discontinued treatment before completion 30. Therefore, our risk model provided a quick glimpse to crude long-term survival weighing to 1-year postoperative mortality.

In conclusion, based on two independent population-based cohorts, this study has demonstrated a considerable gap between the traditionally defined 30-day surgical mortality and the 1-year mortality of patients with solid-organ cancers undergoing curative resection. It highlights a need for a more deliberate and logical preoperative risk assessment in order to address the tendency to underestimate mortality risk and overanticipate survival gain. Our risk stratification model, developed exclusively from the preoperative data, not only predicts 1-year postoperative mortality but also indicates potential survival outcomes in patients with solid-organ cancers.
